# Molecular and structural insights into SARS-CoV-2 evolution: from BA.2 to XBB subvariants

**DOI:** 10.1128/mbio.03220-23

**Published:** 2024-09-16

**Authors:** Hisano Yajima, Tomo Nomai, Kaho Okumura, Katsumi Maenaka, The Genotype to Phenotype Japan (G2P-Japan) Consortium, Keita Matsuno, Jumpei Ito, Takao Hashiguchi, Kei Sato

**Affiliations:** 1 Hokkaido University, Sapporo, Japan; 2 Division of Systems Virology, Department of Microbiology and Immunolog, The Institute of Medical Science, The University of Tokyo, Tokyo, Japan; 3 Tokyo Metropolitan Institute of Public Health, Tokyo, Japan; 4 Tokai University, Kanagawa, Japan; 5 Kyoto University, Kyoto, Japan; 6 Hiroshima University, Hiroshima, Japan; 7 Kyushu University, Fukuoka, Japan; 8 Kumamoto University, Kumamoto, Japan; 9 University of Miyazaki, Miyazaki, Japan; 10 Charles University, Vestec-Prague, Czechia; 1 Laboratory of Medical Virology, Institute for Life and Medical Sciences, Kyoto University, Kyoto, Japan; 2 Laboratory of Biomolecular Science and Center for Research and Education on Drug Discovery, Faculty of Pharmaceutical Sciences, Hokkaido University, Sapporo, Japan; 3 Division of Systems Virology, Department of Microbiology and Immunology, The Institute of Medical Science, The University of Tokyo, Tokyo, Japan; 4 Faculty of Liberal Arts, Sophia University, Tokyo, Japan; 5 Institute for Vaccine Research and Development, HU-IVReD, Hokkaido University, Sapporo, Japan; 6 Global Station for Biosurfaces and Drug Discovery, Hokkaido University, Sapporo, Japan; 7 Division of Pathogen Structure, International Institute for Zoonosis Control, Hokkaido University, Sapporo, Japan; 8 Faculty of Pharmaceutical Sciences, Kyushu University, Fukuoka, Japan; 9 International Research Center for Infectious Diseases, The Institute of Medical Science, The University of Tokyo, Tokyo, Japan; 10 Kyoto University Immunomonitoring Center, Kyoto University, Kyoto, Japan; 11 Graduate School of Medicine, The University of Tokyo, Tokyo, Japan; 12 Graduate School of Frontier Sciences, The University of Tokyo, Kashiwa, Japan; 13 International Vaccine Design Center, The Institute of Medical Science, The University of Tokyo, Tokyo, Japan; 14 Collaboration Unit for Infection, Joint Research Center for Human Retrovirus Infection, Kumamoto University, Kumamoto, Japan; 15 MRC-University of Glasgow Centre for Virus Research, Glasgow, United Kingdom; Albert Einstein College of Medicine, Bronx, New York, USA

**Keywords:** SARS-CoV-2, Omicron, spike, evolution, structural biology, molecular phylogenetic

## Abstract

Due to the incessant emergence of various SARS-CoV-2 variants with enhanced fitness in the human population, controlling the COVID-19 pandemic has been challenging. Understanding how the virus enhances its fitness during a pandemic could offer valuable insights for more effective control of viral epidemics. In this manuscript, we review the evolution of SARS-CoV-2 from early 2022 to the end of 2023—from Omicron BA.2 to XBB descendants. Focusing on viral evolution during this period, we provide concrete examples that SARS-CoV-2 has increased its fitness by enhancing several functions of the spike (S) protein, including its binding affinity to the ACE2 receptor and its ability to evade humoral immunity. Furthermore, we explore how specific mutations modify these functions of the S protein through structural alterations. This review provides evolutionary, molecular, and structural insights into how SARS-CoV-2 has increased its fitness and repeatedly caused epidemic surges during the pandemic.

## INTRODUCTION

The ability of viruses to evolve and change their characteristics causes major challenges in controlling viral infections. The COVID-19 pandemic has been difficult to control due to the emergence of variants with enhanced transmissibility and immune escape ability (1, 2). Through the extensive implementation of viral genomic epidemiology and efforts in the field of basic virology, the evolution of SARS-CoV-2 has been tracked in detail and almost in real time. It is not exaggerating to say that research on the evolution of SARS-CoV-2 has involved the most detailed examinations of viral (or any organism’s) evolution in the history of science. It is anticipated that the principle of viral epidemics and evolution can be elucidated using SARS-CoV-2 as a model virus. We, the G2P-Japan consortium, have been elucidating the characteristics of successively emerging SARS-CoV-2 variants and tracing their evolutionary trajectories using a multidisciplinary approach (3–28).

**Fig 1:**
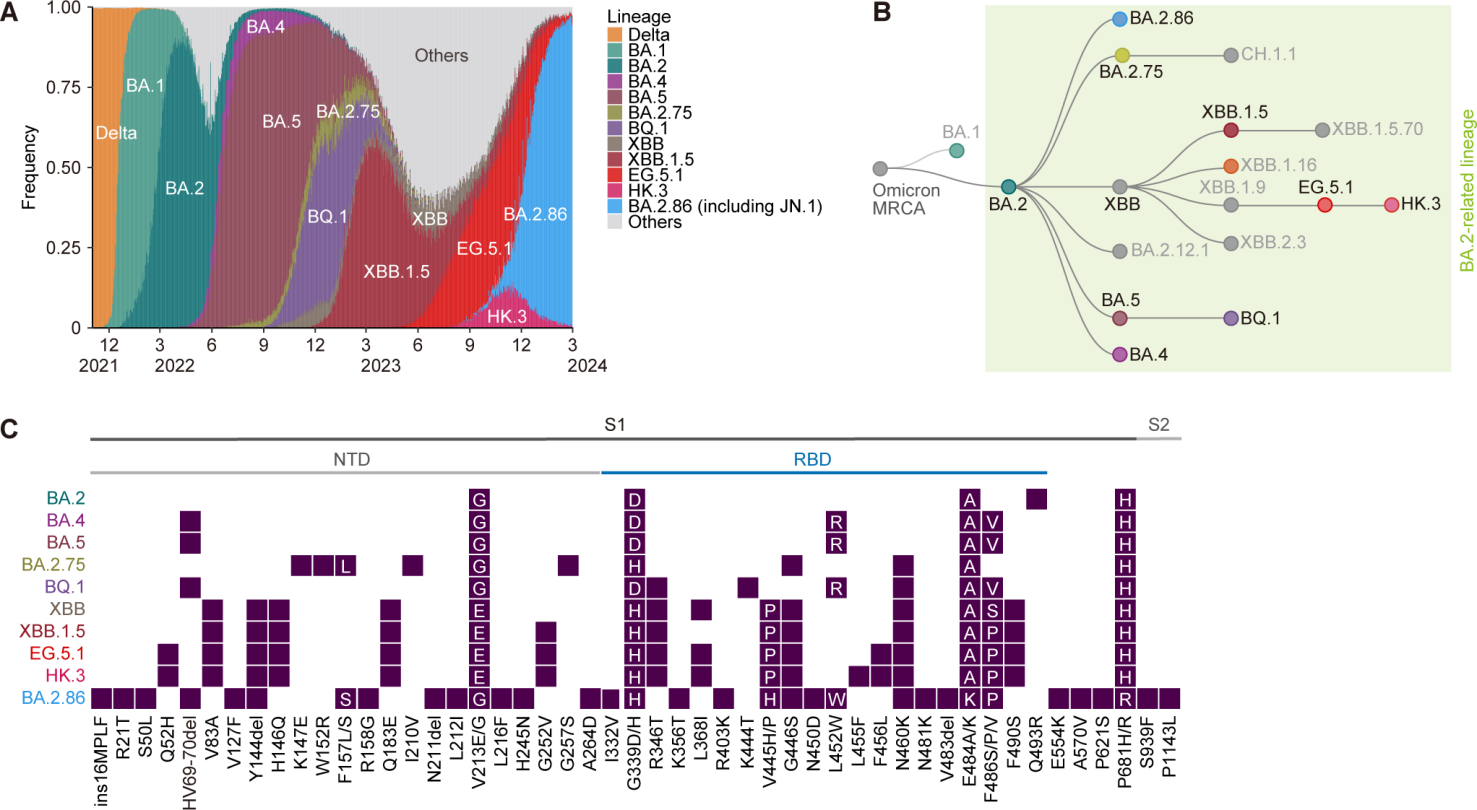
Epidemiology and evolution of BA.2-related lineages. (**A**) Repeated transitions of epidemic SARS-CoV-2 lineages from 1 November 2021 to 29 February 2024. The source data were obtained from GISAID (https://gisaid.org/; EPI SET ID: EPI_SET_240423yq). (**B**) Schematic lineage tree of major epidemic lineages provided by Nextclade. This figure was sourced from Nextclade (https://clades.nextstrain.org/) and used under the CC-BY 4.0 license. (**C**) Comparison of mutations in the S protein among major lineages. When different types of substitutions occur at the same amino acid position, these substitutions are displayed together, indicating the types of substitutions. The source data were obtained from GISAID (EPI SET ID: EPI_SET_240423yq).

Viral fitness, that is, the ability of viruses to spread in the host population, can be compared among variants based on a numerical parameter called the effective reproduction number (R_e_) (29–32). R_e_ represents the average number of secondary infections caused by an infected individual in a certain condition. Throughout the COVID-19 pandemic, every time a new variant with greater fitness has emerged, the prevalent variant has shifted from an existing variant to the new variant, causing new epidemic surges (Fig. 1A).

SARS-CoV-2 undergoes mutations (including substitutions, deletions, and insertions) in viral proteins to enhance its fitness and exhibit different characteristics (31). The spike (S) protein is critical for attachment to receptors, mainly angiotensin-converting enzyme 2 (ACE2), triggering virus entry into target cells (33). In addition, the S protein is the primary target for neutralizing antibodies, which are the major component of the humoral immune response triggered by vaccinations or natural infections (34). Antibodies inhibit binding between the S protein and ACE2 receptor and subsequently prevent viral infection of cells (34). Consequently, mutations in the S protein can affect its binding affinity for the ACE2 receptor and its ability to evade neutralizing antibodies (1, 2, 9, 11, 13–18, 22–24, 26, 28). Newly emerged SARS-CoV-2 variants have acquired mutations that enhance these two functional aspects of the S protein, thereby boosting their fitness (1, 2).

At the end of 2021, a novel SARS-CoV-2 variant, currently called Omicron, was first identified in South Africa (35). Omicron rapidly caused epidemic surges worldwide, virtually eliminating previous variants, such as Delta, from human populations. Three Omicron lineages, BA.1, BA.2, and BA.3, were initially identified. While BA.1 became the first dominant variant globally, the prevalent global variant quickly shifted to BA.2 (11). Subsequently, BA.2 and its related lineages, including BA.4 and BA.5, diversified rapidly, generating numerous variants. From early 2022 to the present, almost all circulating variants are categorized into these BA.2-related lineages (Fig. 1B).

In this paper, we first review the epidemic and evolutionary history of BA.2-related lineages until the end of 2023, including variants from BA.2 to XBB subvariants (informative reviews covering the earlier evolution of SARS-CoV-2 have also been published elsewhere [1, 2]). We then explore the alterations in S proteins that occurred during the evolutionary process from the viewpoint of structural virology. This review provides molecular and structural insights into the evolution of SARS-CoV-2.

## MAIN

### Epidemic and evolutionary history of BA.2-related lineages

#### Spring–summer 2022: emergence of BA.2 to BA.5

By the end of March 2022, BA.2 was almost entirely dominant over other lineages worldwide (Fig. 1A). In this period, BA.4 and BA.5, close relatives of BA.2, were identified in South Africa, the initial epicenter of Omicron (36). It is unclear whether BA.4 and BA.5 are direct descendants or sister lineages of BA.2, but this distinction may not be significant since these lineages arose through recombination events and thus have multiple ancestry pathways (Fig. 1B) (36). BA.4 and BA.5 share identical S proteins, and compared to BA.2, the S proteins of these variants have three amino acid substitutions (L452R, F486V, and R493Q [a revertant]) in the receptor-binding domain (RBD) and a deletion (HV69-70del) in the N-terminal domain (NTD) (Fig. 1C). Both BA.4 and BA.5 demonstrated a greater R_e_ value, approximately 1.3-fold over that of BA.2 in South Africa, leading to their global spread (13). Although both BA.4 and BA.5 spread worldwide, BA.5 eventually became more prevalent, presumably due to its slightly higher R_e_ (Fig. 1A).

#### Summer 2022: emergence of BA.2.75, a “second generation of BA.2”

Although BA.5 is dominant globally, several descendants of BA.2 with a relatively higher number of mutations, including BA.2.75 and BJ.1, have been identified primarily in South Asian countries such as India. These variants are referred to as “second-generation BA.2.” Identified in May 2022 in India, BA.2.75 has nine amino acid substitutions in the S protein compared to BA.2 (five mutations in the NTD; K147E, W152R, F157L, I210V, and G257S and four mutations in the RBD; D339H, G446S, N460K, and R493Q) (Fig. 1C) (14). BA.2.75 exhibited approximately 1.35-fold greater R_e_ than that of BA.2 and became dominant in India, where BA.2 was prevalent at the time. However, this variant did not spread extensively in regions where BA.5 was already dominant, presumably because the R_e_ of BA.2.75 was not substantially greater than that of BA.5 (approximately 1.1-fold) (Fig. 1C).

#### Autumn–winter 2022: formation of “variant soup,” including BQ.1.1 and XBB lineages

Following the global epidemic surges driven by the BA.5 variant, a series of variants exhibiting similarly high Rₑ values emerged sporadically across different regions. As a result, an epidemiological scenario occurred late in 2022 in which multiple variants coexisted without a single dominant variant, which has often been referred to as “variant soup” (37). Notably, these variants convergently acquired substitutions at specific amino acid residues, such as R346, L452, K444, N460, and F486 (16). Of these variants, BQ.1.1 (BA.5.3.1.1) and XBB are noteworthy. BQ.1.1 is a descendant of BA.5 that arose through the sequential acquisition of the substitutions K444T, N460K, and R346T and was first identified in Africa and Europe (Fig. 1C) (16). On the other hand, XBB was detected first in South Asia and is a variant that emerged through a recombination event in the RBD of the S protein between two second-generation BA.2 variants, BA.2.75 (more specifically, BM.1.1.1; BA.2.75.3.1.1.1) and BJ.1 (17). Due to this recombination, XBB has a total of 14 mutations compared to BA.2 (five mutations in the NTD and nine mutations in the RBD). Both BQ.1.1 and XBB showed a greater R_e_, approximately 1.25-fold that of BA.5, leading to a shift in the prevalent variants from BA.5 to these new variants (17). BQ.1.1 was predominant in Western countries, while XBB was predominant in Eastern countries, corresponding to their respective regions of origin.

#### Winter 2022–spring 2023: emergence of XBB.1.5

The cocirculation or equilibrium of BQ.1.1 and XBB continued until the end of 2022, but it was disrupted by the emergence of XBB.1.5 (15). XBB.1.5, which acquired the F486P substitution, is a descendant of XBB and rapidly spread in the United States from the end of November 2022 (Fig. 1C). Originally, XBB carried the F486S substitution (one nucleotide substitution from BA.2), but in XBB.1.5, the Serine at position 486 of XBB was further substituted with proline (one nucleotide substitution from XBB; that is, two nucleotide substitution from BA.2). By acquiring the F486P substitution, XBB.1.5 showed an R_e_ approximately 1.2-fold greater than those of XBB.1 and BQ.1.1 (15). Consequently, XBB.1.5 spreads globally, causing larger epidemic surges (Fig. 1A). In addition to XBB.1.5, multiple XBB descendants with higher R_e_ also emerged, and each descendant convergently acquired F486P (18). This observation underscores the importance of this substitution for enhanced viral fitness.

#### Spring–summer 2023: emergence of EG.5.1

Until the summer of 2023, various descendant lineages of XBB that acquired the F486P mutation coexisted and circulated. The next variants with significantly increased prevalence were variants that acquired F456L substitutions in addition to F486P. Convergent emergence of the XBB sublineage with F456L occurred in EG.5.1 (XBB.1.9.2.5.1), which possesses F486P, F456L, and Q52H in comparison to XBB.1 (Fig. 1C). The R_e_ of EG.5.1 was approximately 1.2-fold greater than that of XBB.1.5, and it notably spread across East or Southeast Asia and North America (38).

#### Summer 2023–winter 2024: emergence of “FLip” variants, including HK.3

Subsequently, XBB sublineages bearing both F486P and F456L, such as EG.5.1, further evolved by acquiring the L455F substitution in a convergent manner. These variants, in which the amino acid group LF is swapped to FL at positions 455 and 456, are referred to as “FLip” variants (Fig. 1C) (39). Notably, HK.3 (EG.5.1.1.3; XBB.1.9.2.5.1.1.3), a descendant of EG.5.1 identified first in China, demonstrated the highest R_e_ value among the XBB lineage variants (24).

#### Summer 2023–summer 2024: emergence of BA.2.86 variants, including JN.1

In the summer of 2023, BA.2.86, which is phylogenetically distinct from the XBB lineages, emerged from BA.2 by acquiring approximately 30 mutations in the S protein and gradually began spreading worldwide (23). Notably, because BA.2.86 exhibited an R_e_ nearly equivalent to that of HK.3, HK.3 remained prevalent across multiple regions. At the end of 2023, the XBB lineages were almost completely displaced from the human population by JN.1, a descendant of BA.2.86 with the L455S substitution (Fig. 1A) (26).

As of July 2024, JN.1 (BA.2.86.1.1) has further diversified, resulting in the emergence of various descendant variants such as KP.2 (BA.2.86.1.1.11.1.2; JN.1.11.1.2) and KP.3 (BA.2.86.1.1.11.1.3; JN.1.11.1.3). Importantly, the previously mentioned R346T and F456L substitutions have been repeatedly acquired in these JN.1 descendant variants. This observation highlights the evolutionary significance of these substitutions in BA.2.86 as well.

### Molecular and structural basis of SARS-CoV-2 evolution

The evolutionary history of the SARS-CoV-2 Omicron variants until late 2023 and the mutations acquired by the respective variants are described above (Fig. 1). Hereafter, we describe the impact of various mutations on the structure and phenotype of SARS-CoV-2 S proteins, with a focus on several of our studies.

#### Divergent evolution from BA.2 to BA.5 and BA.2.75

**Fig 2:**
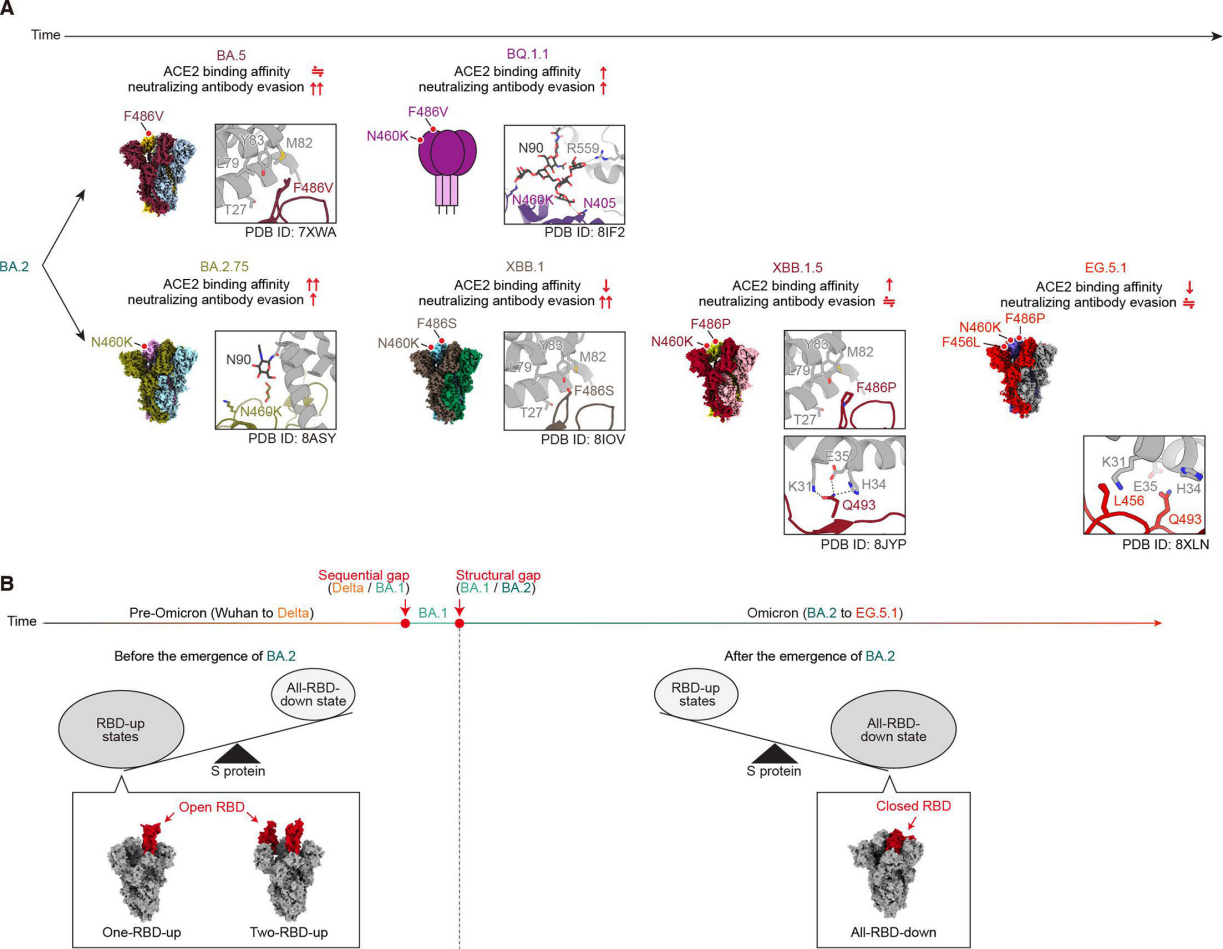
Structural and virological summaries of the evolution of BA.2-related lineages. (**A**) A schematic of descendants after BA.2 was sorted over time (refer to Fig. 1A). The ACE2-binding affinity and neutralizing antibody evasion ability compared to those of the ancestral lineage on the left are shown under each variant name. In each variant, the cryo-EM structure or schematic of the S protein trimer alone is shown in the bottom left, and the zoom-up view of the mutation of interest for each variant is shown in the bottom right. PDB IDs: BA.5, 7XWA; BA.2.75, 8ASY; BQ.1.1, 8IF2; XBB.1, 8IOV; XBB.1.5, 8JYP; EG.5.1, 8XLN. EMDB IDs: BA.5, 33325; BA.2.75, 34221; XBB.1, 35622; XBB.1.5, 36724; EG.5.1, 37651. (**B**) A schematic diagram of the sequence gap bordered before/after BA.1 and the structural gap bordered before/after BA.2. In sequences, the difference between pre-Omicron and Omicron is obvious, but in the RBD up/down states of the S proteins, they are separated at the boundary of BA.1 and BA.2. In the structures of S-protein trimer, the RBDs are highlighted in red (the other areas are gray).

To reveal the virological characteristics of the BA.5 lineage, we characterized its ability of ACE2 binding and neutralizing antibody evasion as well as the X-ray crystal structure of the RBD–ACE2 complex (13). In the BA.5 lineage, the F486V substitution in the RBD is responsible for evading neutralizing antibodies, as demonstrated by assays using human sera and pseudoviruses bearing the BA.2-based S protein harboring the F486V substitution. However, F486V decreased the binding affinity for the ACE2 receptor. To compensate for the decreased binding affinity, which may be disadvantageous to the survival strategy of BA.5, the variant has also acquired L452R; this mutation was also observed in Delta that enhances ACE2-binding affinity (3). Consequently, by acquiring three mutations in the RBD, BA.5 maintained an ACE2-binding affinity equivalent to that of BA.2. F486 of the SARS-CoV-2 S protein is a dominant epitope of broadly neutralizing antibodies against various variants (40, 41), indicating that mutation of this residue provides a significant advantage in the survival strategy of BA.5. Therefore, BA.5 was characterized as the first variant in which the amino acid residue 486 was mutated to enhance the ability to evade neutralizing antibodies (Fig. 2A).

To clarify the virological characteristics of the BA.2.75 lineage, we also reported the ACE2-binding affinity and neutralizing antibody evasion as well as cryo-EM structures of the S protein alone and the S trimer–ACE2 complex (14, 42). In the BA.2.75 lineage, the N460K mutation was shown not only to significantly enhance ACE2 receptor-binding affinity as demonstrated by a yeast surface display assay using the SARS-CoV-2 S RBD and soluble human ACE2 but also to increase neutralizing antibody evasion ability by assays utilizing human sera and pseudoviruses bearing point-mutated S proteins. Notably, the structural analysis suggested that the N460K of the BA.2.75 S protein formed an electrostatic interaction with the N90-linked glycan on ACE2. Point mutation experiments with both the ACE2 and BA.2.75 S proteins showed that the interaction between the N460K of the BA.2.75 S and the N90-linked glycan on ACE2 positively contributes to S-ACE2 binding (43). Previous variants predating BA.2.75 have been reported to exert inhibitory effects on SARS-CoV-2 infection through the N90-binding glycan on ACE2 (13). Accordingly, BA.2.75 can be assigned as the branching-point variant that evolved in favor of the N90-linked glycan on ACE2, which had previously been an inhibitory factor of cell entry and was advantageous for survival (Fig. 2A).

#### Convergent evolution leading to BQ.1 and XBB lineages

To determine the virological characteristics of the BQ.1 lineage, we reported the ACE2-binding affinity and neutralizing antibody evasion as well as the X-ray crystal structure of the RBD–ACE2 complex (16). In the BQ.1 lineage, similar to BA.5, F486V plays an important role in neutralizing antibody evasion; however, the ACE2-binding affinity is reduced by F486V. To compensate for the reduced ACE2-binding affinity, BQ.1.1 acquired N460K, the mutation that first arose in BA.2.75. In the study focusing on BQ.1.1, the interaction between N460K of the S protein and the N90-linked glycan on ACE2 was reported by X-ray crystallographic analysis (16). N460K of the S protein, together with the amino acid residues N405 of the S protein and R559 of ACE2, formed an interaction network with N90-linked glycan on ACE2. This glycan-mediated interaction network stabilized the binding between ACE2 and the S RBD. Hence, BQ.1.1 represents a convergent evolutionary variant in which the dual effects of F486V and N460K, combined with BA.5 and BA.2.75, enhanced ACE2-binding affinity and neutralizing antibody evasion, resulting in increased viral fitness (Fig. 2A).

To elucidate the virological characteristics of XBB.1, we also characterized its ACE2 binding and neutralizing antibody evasion as well as cryo-EM structures of the S protein and the S trimer–ACE2 complex (17). XBB.1 acquired a mutation at amino acid residue 486 of the S protein, similar to BA.5 and BQ.1.1; however, the mutations in BA.5 and BQ.1.1 were the hydrophobic Valine, while the mutation in XBB.1 was the hydrophilic Serine. The residue 486 of the SARS-CoV-2 S protein interacts with the hydrophobic patch composed of amino acid residues F28, L79, M82, and Y83 of ACE2. Therefore, the residue 486 of the XBB.1 S was substituted into a hydrophilic serine residue, which is unfavorable for interaction with ACE2; thus, the ACE2-binding affinity was significantly reduced compared to that of the ancestral variant, BA.2.75. By contrast, F486S significantly enhanced the ability of XBB.1 to evade neutralizing antibodies. Consequently, XBB.1 was also a convergent evolutionary variant that acquired the F486S and N460K combinatorial mutation and increased viral fitness (Fig. 2A).

Overall, the SARS-CoV-2 omicron lineage diverged once from BA.2 into BA.5 and BA.2.75 lineages characterized by F486V and N460K, respectively. Subsequently, BA.5 and BA.2.75 evolved into the BQ.1 and XBB lineages, which acquired F486X and N460K mutations, respectively. F486S, which was acquired by XBB.1, reduced the binding affinity of ACE2, whereas the ability to evade neutralizing antibodies was significantly enhanced. Notably, XBB.1 became a remarkable variant that spread through Asia despite exhibiting reduced ACE2-binding affinity than that of BA.2.75. Subsequently, XBB.1.5, which acquired only a single proline mutation at position 486 of the S protein, emerged (15). The F486P mutation allowed XBB.1.5 to form a hydrophobic interaction, as revealed by cryo-EM structures, between P486 in the S protein RBD and the hydrophobic patch consisting of the F28, L79, M82, and Y83 residues of ACE2 (27). Compared to XBB.1, XBB.1.5 maintained an equivalent ability to evade neutralizing antibodies and the overall structure of the S protein but exhibited increased ACE2-binding affinity, resulting in increased viral fitness (Fig. 2A).

#### Evolutionary strategy of the XBB lineage leading to EG.5.1 and HK.3

To determine the virological characteristics of the EG.5.1 linage, we characterized its ACE2-binding ability and neutralizing antibody evasion as well as the cryo-EM structures of the S protein and the S trimer–ACE2 complex (38). In the EG.5.1 lineage, the characteristic F456L mutation in the S RBD reduced ACE2-binding affinity. The cryo-EM structure of the RBD–ACE2 complex showed that F456L of the S protein interacts with K31 of ACE2 at the interface. In the case of XBB.1.5, K31, H34, and E35 of ACE2 formed a hydrogen-bond network with Q493 of the XBB.1.5 S RBD. On the other hand, in the case of EG.5.1, F456L allows for closer proximity to K31 of ACE2, thereby disrupting the hydrogen bond network around Q493 of the EG.5.1 S RBD. Indeed, the ACE2-binding affinity of the EG.5.1 S is significantly lower than that of the XBB.1.5 S. In addition, in the RBD-closed state of the EG.5.1 S trimer, F456L, which is located at the interface with the adjacent protomer, weakened the hydrophobic interaction with P373 of the adjacent RBD and loosened the RBD packing in an S trimer conformation. This conformation facilitated the transition to the one RBD-up state, suggesting that the conformation compensated for the negative effect on the weaker ACE2-binding affinity. By contrast, the cell-to-cell fusion activity of the EG.5.1 S protein was comparable to that of the XBB.1.5 S protein, suggesting that the change in cell-to-cell fusion activity, which compensated for the reduced ACE2-binding affinity, was caused by the conformational transition of the S protein trimer. The membrane fusion activity is defined as the sum of ACE2 receptor binding and membrane fusion. In a neutralization assay with XBB breakthrough infection sera, a pseudovirus bearing the EG.5.1 mutation F456L showed significantly enhanced neutralizing antibody evasion ability compared to that of XBB.1.5 (22). Compared with XBB.1.5, we and others reported that HK.3, which acquired the L455F mutation in addition to F456L, referred to as FLip above, helped increase the ability to evade neutralizing antibodies (22, 44). Altogether, the XBB lineage, which appeared to have converged and evolved by acquiring two characteristic mutations, N460K and F486P, evolved further by acquiring the FLip mutation (Fig. 2A). In this context, it might be reasonable to assume that new lineages appear when the convergence of mutations reaches a plateau in a sublineage—for example, JN.1, a subvariant of BA.2.86, has more than 30 mutations in the S protein and outcompeted XBB subvariants including HK.3 (23).

## CONCLUSION AND FUTURE PERSPECTIVE

The fitness (R_e_) of SARS-CoV-2 increased through mutations during its evolution, leading to repeated epidemic surges. Variants with significantly increased fitness possess mutations that boost immune evasion and/or the ability to bind to ACE2, highlighting the critical role of these mutations in enhancing fitness. Previous studies, including ours, have adopted an approach that directly models the relationship between viral mutations and fitness, demonstrating that these mutations contribute to an increase in fitness (16, 31, 45, 46). Moreover, these mutations were acquired in a convergent manner throughout the evolution of SARS-CoV-2, emphasizing their evolutionary importance. Hie et al. proposed that viral proteins evolve by altering antigenicity while retaining their original function to evade existing humoral immunity (47). According to the evolutionary pattern described in this review, it appears that SARS-CoV-2 proteins, particularly the S proteins after the emergence of Omicron BA.2, are evolving in accordance with this rule.

As for changes before and after Omicron, Omicron BA.1 emerged with more than 30 mutations in the S protein and dramatically reduced ability to infect lung tissue (48, 49) while exhibiting an increased capacity for nasal tissue infection (49). This tropism shift was also retained in BA.2 and its more recent descendants—the BQ.1 and XBB lineages (49)—while BA.5 might regain tropism to the lung tissues (50). This fact demonstrates that the accumulation of mutations can cause shifts in cellular tropism even when the use of ACE2 receptors remains similar.

Based on the experimental results, the following mutations are notable in convergent evolution: F486V/P/S and N460K (13, 16, 17, 27, 38). While F486V/P/S led to a lower ACE2-binding affinity, it substantially enhanced neutralizing antibody evasion. Residue 486 of the S protein has been reported as a key epitope for broadly neutralizing antibodies in some structural analyses (51). By contrast, N460K resulted in a significantly greater ACE2-binding affinity and contributed to enhanced ability to evade neutralizing antibodies. The large change in the electrostatic potential of the molecular surface of the S protein from neutral Asparagine to positively charged Lysine and the novel interaction owing to the proximity of the N90-linked glycan on ACE2 are the structural basis for this evolution caused by the N460K mutation (43). The lineages BQ.1 and XBB, harboring both F486V/P/S and N460K mutations, have been regarded as crucial branching points in the subsequent evolution of SARS-CoV-2.

By examining the evolution from the SARS-CoV-2 Omicron lineage BA.2 to EG.5.1 using molecular and structural findings, we observed a trend in which globally prevalent variants, such as the BA.5 and XBB lineages, exhibited a substantially enhanced ability to evade neutralizing antibodies rather than a greater ACE2-binding affinity compared to their parental/ancestral variant (13, 16, 17, 27, 38). While the F486V/P/S and N460K mutations play pivotal roles in SARS-CoV-2 infectivity, variations in the overall structure of the S protein were also observed among the different variants. In the pre-Omicron variants—such as the ancestral Wuhan strain, the D614G strain, Alpha, and Delta—the S protein structures were dominated as RBD-up states, especially owing to D614G (52), although all-RBD-down states were also observed to some extent (Fig. 2B) (53–56). Interestingly, the RBD-up states were also predominant in Omicron BA.1 (56–61). Therefore, one-RBD-up state predominates for S proteins in the pre-Omicron variants and BA.1 (Fig. 2B). However, after the emergence of Omicron BA.2, all-RBD-down states have shifted to predominance (Fig. 2B) (62, 63). Although the amino acid sequences of SARS-CoV-2 S protein have changed dramatically after the emergence of Omicron BA.1, the states of RBD conformation (i.e., RBD-up or RBD-down) were maintained even in Omicron BA.1 and largely shifted after the emergence of BA.2 (Fig. 2B). The open RBD form can be a primary target of neutralizing antibodies, and therefore, the conformational shift to the closed RBD form, hiding the ACE2-binding site, may be a strategy of BA.2’s to evade neutralizing antibodies. However, it should be noted that the proportion of the RBD at up/down states depends, in part, on the expression conditions (e.g., the sequences of the expression plasmid used and the condition for structural analysis). Therefore, we herein discuss the general trend of the states of RBD conformation (i.e., RBD-up or RBD-down). After BA.2.75 emerged (14), two all-RBD-closed states (closed-1 and closed-2) in the S protein were observed and the ratio of the one-RBD-up structure in the S protein was also shifted for each variant (14, 17, 27, 38, 64, 65). Altogether, the overall structures, especially RBD up/down conformations, of the S proteins gradually shifted, even in the newly emerged variants involving only a few amino acid substitutions (14, 17, 27, 38, 63–65).

This review discusses a limited range of evolution from the BA.2 to the XBB lineage based on structural and molecular experimental data, but SARS-CoV-2 likely continues to evolve and alter the structures of the S protein. In addition to the humoral immunity (i.e., neutralizing antibodies) targeting the S protein, intrinsic/innate immunity against SARS-CoV-2 infection may also play a pivotal role in driving SARS-CoV-2 evolution, especially the S protein-mediated cell entry process (66). In particular, the human interferon-induced transmembrane protein (IFITM) family reportedly inhibits cell entry in several viruses, including SARS-CoV-2 (66), and SARS-CoV-2 evolution influences IFITM sensitivity (67). Mechanistic studies of IFITM1/2/3 suggest that these factors inhibit the fusion between the viral envelope and cell membrane by increasing cell membrane rigidity while decreasing its fluidity (68, 69). Other intrinsic inhibitors (66) of SARS-CoV-2 entry include such as cholesterol 25-hydroxylase (CH25H) causing membrane modulation (70), lymphocyte antigen 6E (LY6E) leading receptor downmodulation or endosome redirection (71), CD74 inhibiting cathepsin activity (72), and hepatocyte growth factor activator inhibitor 2 (HAI-2) inhibiting TMPRSS2 activity (73). Intrinsic inhibitors would also contribute to the evolution of SARS-CoV-2 adapted to the human population. Accordingly, the findings here are expected to contribute to the identification of noteworthy SARS-CoV-2 variants in the future and countermeasures against these variants.

In conclusion, surveilling viral genomes (i.e., genotype) and elucidating the virological and structural features of newly emerged, highly transmissible SARS-CoV-2 variants (i.e., phenotypes) are critical to understanding the evolution of SARS-CoV-2. Studies on the evolution of SARS-CoV-2 will provide insights that contribute to developing more efficient infection control methods and new vaccine developments.
